# The effects of crank power and cadence on muscle fascicle shortening velocity, muscle activation and joint-specific power during cycling

**DOI:** 10.1242/jeb.245600

**Published:** 2023-07-12

**Authors:** Cristian D. Riveros-Matthey, Timothy J. Carroll, Glen A. Lichtwark, Mark J. Connick

**Affiliations:** School of Human Movement and Nutrition Sciences, Centre for Sensorimotor Performance, The University of Queensland, Brisbane, QLD 4067, Australia

**Keywords:** Vastus lateralis muscle, Joint power, Crank power requirements, Muscle mechanics, Electromyography

## Abstract

Whilst people typically choose to locomote in the most economical fashion, during bicycling they will, unusually, chose cadences that are higher than metabolically optimal. Empirical measurements of the intrinsic contractile properties of the vastus lateralis (VL) muscle during submaximal cycling suggest that the cadences that people self-selected might allow for optimal muscle fascicle shortening velocity for the production of knee extensor muscle power. It remains unclear, however, whether this is consistent across different power outputs where the self-selected cadence (SSC) varies. We examined the effect of cadence and external power requirements on muscle neuromechanics and joint power during cycling. VL fascicle shortening velocity, muscle activation and joint-specific power were measured during cycling between 60 and 120 rpm (including SSC), while participants produced 10%, 30% and 50% of peak maximal power. VL shortening velocity increased as cadence increased but was similar across the different power outputs. Although no differences were found in the distribution of joint power across cadence conditions, the absolute knee joint power increased with increasing crank power output. Muscle fascicle shortening velocity increased in VL at the SSC as pedal power demands increased from submaximal towards maximal cycling. A secondary analysis of muscle activation patterns showed minimized activation of VL and other muscles near the SSC at the 10% and 30% power conditions. Minimization of activation with progressively increasing fascicle shortening velocities at the SSC may be consistent with the theory that the optimum shortening velocity for maximizing power increases with the intensity of exercise and recruitment of fast twitch fibers.

## Teaser

**Summary:** Shortening velocity at self-selected cadence increases with power output, whereas at low power demands, minimizing knee muscle activation becomes important in cadence selection during cycling.

## INTRODUCTION

Humans tend to locomote in ways that minimize energy consumption (Bertram, 2005; Ralston, 1958; Hoyt, 1981; Zarrugh et al., 1974; Alexander, 1989, 2000; Minetti and Alexander, 1997; Snaterse et al., 2011; Sparrow and Newell, 1998). A good example of this is during walking or submaximal running (i.e. below lactate threshold), where people tend to locomote at a step frequency near that which minimizes the metabolic energy cost. However, people do not show the same behavior when riding a bicycle, and tend to use higher cadences than the metabolically optimal one (Brisswalter et al., 2000; Lucia et al., 2000; Marsh and Martin, 1997; Brennan et al., 2019). Apparently, other criteria besides energetic economy are at play in the self-selected pedaling rate during cycling.

Several alternative factors have been proposed to influence cadence selection in cycling (Marsh et al., 2000; Brennan et al., 2019). For example, factors such as muscle activation (Marsh and Martin, 1995; MacIntosh et al., 2000; Neptune et al., 1997; Sarre et al., 2003; Takaishi et al., 1996; Baum and Li, 2003) and net joint moments (Neptune and Hull, 1999b; Hull and Gonzalez, 1987; Marsh et al., 2000) were reportedly minimized at high cadences under submaximal conditions in experimental and simulated environments. Additionally, by exploring the amount of muscle activation per pedaling cycle, studies have shown a minimization of electromyography root mean square (EMG RMS) between 80 rpm (*in vivo*) (Takaishi et al., 1996) and 90 rpm (*in silico*) (Neptune and Hull, 1999b), arguing that these responses are linked to a reduction in muscle fatigue (Hagberg et al., 1981; Takaishi et al., 1998). Importantly, the cadence at which EMG per cycle is minimized shifts towards higher values as power output increases (MacIntosh et al., 2000). However, these protocols were tested under fixed cadence conditions, and the self-selected cadence (SSC) was not determined. Moreover, the study conducted by Marsh et al. (2000), which did include a SSC protocol, found only a modest association between the cadence selected by cyclists and the cadence that minimized a joint moment-based cost function. Thus, it remains unclear whether EMG and joint moment variables are the main drivers of the SSC.

A third mechanical factor that has recently drawn attention is related to the intrinsic contractile properties of the muscles. The ability of muscles to generate force is critically influenced by the length and velocity of muscle fibers, factors that ultimately constrain the capacity of muscles to produce power during cycling. In this context, force–length and force–velocity properties and their mechanical function during cycling under sub-maximal conditions have been reported for the vastus lateralis (VL) muscle, which is a major contributor to cycling power (Austin et al., 2010; Muraoka et al., 2001; Brennan et al., 2018; 2019). For example, Muraoka et al. (2001) found that the timing of fascicle shortening differed from that of the muscle–tendon unit (MTU), because of interaction with the elastic tendinous tissue in series with fibers. Although these observations were made at low cadences and power outputs, they suggest that the elasticity of the VL tendinous tissue decouples fascicle shortening from that of the MTU during force development, specifically the first half of the positive phase of the pedaling cycle. In the same line, Brennan and colleagues (2018), through ultrasound imaging, demonstrated that fibers operate close to their optimal length (*L*_O_) for maximal force production and velocity (*V*_O_) to achieve maximum power output at different cadences (range 40–100 rpm). They found that while VL fascicle shortening velocity increased with cadence, there was a plateau in average shortening velocity at high cadences. Additionally, they found that the operating velocity of the fascicles at the preferred cycling cadence was approximately equal to that predicted to maximize power according to the force–velocity relationship during maximum voluntary contractions (Brennan et al., 2019). These findings provide a deeper understanding of how the muscle properties interact in a range of cadence conditions; however, they are limited by the fact that a single power output condition was assessed (2.5 W kg^−1^).

As SSC increases with increased power output (Hansen et al., 2002; Vogt et al., 2008; Pugh, 1974), there is an increase in muscle force and muscle activation requirements (MacIntosh et al., 2000) and presumably an increase in muscle fascicle shortening velocity to maintain the higher cadence. Both Sargeant (1994) and MacIntosh et al., (2000) have suggested that as power requirements increase, the increased force demand (or resistance) requires recruitment of a greater proportion of fast-twitch fibers according to Henneman's size principle (Henneman et al., 1965), and thus the cadence at which maximum power can be generated also progressively increases. There is some evidence showing an increase in the velocity at which maximum power is generated with increasing recruitment of human muscle (Chow and Darling, 1999). It therefore remains in intriguing possibility that shifts in preference to higher cycling cadences with increasing power demands may be related to shifts in the optimum velocity for generating power.

With varying cadence and external power requirements, there may also be a change in the power-generating distribution between joints. For example, at submaximal conditions (80–340 W), the knee extensors contribute the greatest proportion of total lower limb power, with smaller contributions from the knee flexor and hip extensor (Ericson, 1988; Bini et al., 2010). Conversely, during cycling at near-maximal power output (350–850W), there is a shift in the joint power distribution of the lower limb, such that the hip extensor contribution to crank power is twice the knee extensor joint power contribution, and the knee flexor contribution becomes equal to the knee extensor contribution (Elmer et al., 2011; McDaniel et al., 2014). These responses may be influenced by the constraint of knee extensors to generate power in those conditions (e.g. due to high shortening velocities), thus triggering a redistribution of the power mainly driven by the hip. However, the question of whether muscle shortening velocity constrains muscle power capacity at high power requirements remains unexplored.

The aim of this study was to describe how muscle mechanics and joint-specific power vary with crank power and pedaling cadence during cycling and to determine how fascicle shortening velocity at the self-selected cadence changes with increasing power output. We used ultrasound to measure muscle fascicle length and velocity changes in the VL muscle, and instrumented pedals and motion capture to calculate joint power by inverse dynamics. The extent to which the optimal fascicle shortening velocity for power production shifts with increasing cycling power is difficult to quantify, because of variation in fiber type distribution and motor unit recruitment patterns between muscles and individuals. Thus, observations ranging from no change to modest increases in the VL fascicle shortening velocity at the SSC could be consistent with a role for shortening velocity in driving cycling cadence preferences. Alternatively, other factors such as minimizing muscle activation through joint power redistribution might be critical in cadence selections. To distinguish between these possibilities, we examined the joint power distribution and work done by the lower limb to document the relative contributions of hip, knee and ankle joints across the different external power requirements. We also measured electromyographic activity (EMG) from gluteus maximus (GM), VL, rectus femoris (RF), biceps femoris long head (BFlh) and semitendinosus (ST) to assess the interaction between VL muscle fascicle behavior/joint power and the activation of leg muscles.

## MATERIALS AND METHODS

### Subjects

Twelve healthy men from the university community and cycling sports clubs (mean±s.d., age 27±9.9 years, mass 70±7.9 kg, height 1.76±0.054 m) participated in this study. We recruited level 3–4 cyclists, who are daily riders with a considerable history of involvement with the discipline. The participants gave their written informed consent, and all procedures were approved by the Human Research Ethics Committees (HRECs) of the University of Queensland.

All cycling was performed on a bicycle ergometer (Excalibur Sport, Lode BV, Groningen, The Netherlands) that was adjusted according to the anthropometric characteristics of each subject. The saddle height was normalized to 100% of the greater trochanter length (Bini et al., 2014). The trunk angle was standardized according to the preference of each subject within the range 35–45 deg, with the hands placed on the handlebar drops. The angle of the trunk was defined by the line that connects the anatomical landmarks of the acromion and greater trochanter with respect to horizontal. Visual feedback was used to ensure that posture was maintained during the experimental sessions. The crank length was set to 175 mm. Participants wore cleated shoes which clipped into the pedals (SH-R070 and SH-R540, Shimano, Osaka, Japan).

### Experimental design

**Fig. 1.**
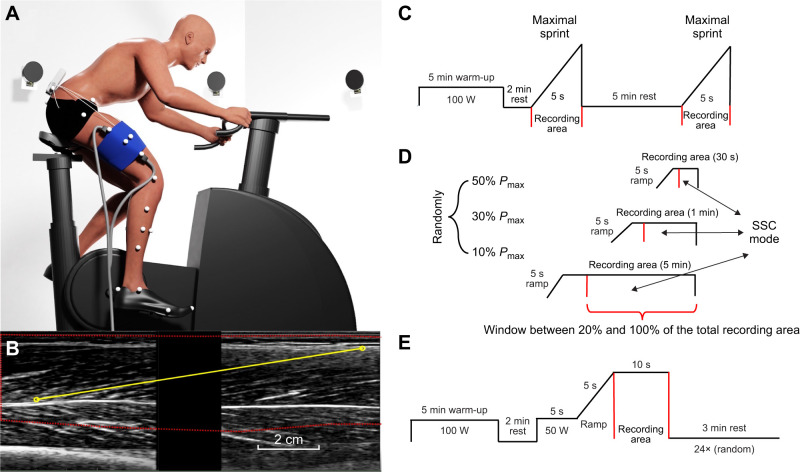
**Experimental setup.** (A) Schematic representation of the experimental design, specifically associated with the day two assessment, with data collected from 3D motion capture, ultrasound of the vastus lateralis (VL) muscle, surface electromyography (EMG) and radial tangential force on the cycle ergometer. (B) Estimating fascicle length in VL muscle through dual probe images during cycling. (C–E) Illustration of the experimental protocol, which was divided into two assessment days. Day one: force–velocity test (C) and self-selected cadence (SSC) protocol (D). Day two: main cycling protocol (E).

Each participant completed two assessment sessions on different days. The assessment on day one was divided into three different parts. The first part comprised the force–velocity test, in which the maximum power that the participant could exert on the ergometer cranks (*P*_max_) was measured (Fig. 1C). This test involved two trials, in each of which the participant performed a maximal sprint of 5 s with a resistance of 1 N m kg^−1^ body mass, followed by a rest period of 5 min (Dorel et al., 2010, 2005; Morin and Samozino, 2019). Participants were verbally encouraged to perform maximally during each trial. In the second part, the self-selected cadence of each participant was determined at three different intensities (10%, 30% and 50% of the *P*_max_). These tests lasted for 5 min, 1 min and 30 s for the 10%, 30% and 50% intensities, respectively (Fig. 1D). Finally, in the third part, participants underwent a familiarization of the cadence-matching protocol to be applied on day two. Each participant completed 6 bouts of cycling at cadences that were randomly selected from 60 to 120 rpm, and at power outputs that were randomly selected from 10%, 30% and 50% of the *P*_max_.

In the assessment on day two, each subject cycled at three different percentages of the *P*_max_ obtained in the force–velocity test described above. For each power level, participants were asked to cycle at eight different cadences (60, 70, 80, 90, 100, 110 and 120 rpm and the self-selected cadence), resulting in a total of 24 trials (Fig. 1E). Before the session, participants began with a 5 min warm-up at 100 W at a SSC. For each trial, they were asked to cycle for approximately 20 s. For the first 5 s, riders pedaled at 100 W at the nominated cadence. Then, for the remaining 15 s, participants were exposed to a 5 s incremental ramp in crank resistance until the desired power output was reached. At this intensity level, their behavior was recorded for 10 s. Each trial concluded with active recovery at 100 W for 3, 2 or 1 min depending on the previous intensity (50%, 30%, 10% of the *P*_max_) (Wilkinson et al., 2020). The following measurements were taken during each trial: 3D motion capture, surface EMG, ultrasound of the VL muscle, radial tangential force, and pedal angle on the cycle ergometer (Fig. 1A).

### Instruments

#### Kinematics

Lower limb kinematics were recorded at 200 Hz using 14 3D motion-capture cameras (Oqus, Qualisys, AB, Gothenburg, Sweden) and software (Qualisys Track Manager). Twenty-two small light-weight reflective markers were attached to the participant's skin. Markers were placed in rigid clusters of three on the mid-thigh and mid-shank, and individually on the anterior and posterior iliac spines, greater trochanter, medial and lateral epicondyles, medial and lateral malleoli, calcaneus, and first and fifth metatarsal heads. After placement of the reflective markers on each cyclist, a trial was captured in a static standing position with the arms crossed. The static recording was then used for scaling purposes during data analysis. To create a global coordinate system, reflective markers in the ergometer cycle were placed on the back of the saddle and at the left and right rear support angle of the cycle ergometer. The cameras tracked the 3D trajectories from each marker at a 200 Hz sampling rate.

#### External forces

We used a wireless, instrumented crank device (Axis, SWIFT Performance, Brisbane, QLD, Australia) to measure the orthogonal crank forces in polar coordinates, crank torque (tangential force×length), and the crank axial force (radial force). From these measurements, the vertical (**F***_z_*) and horizontal (**F***_x_*) components of pedal forces were derived. Force data were synchronously sampled via an analog to digital (A/D) board capturing at 100 Hz via the Qualisys Track Manager Software. Before each assessment, dynamic and static calibrations were performed on the instrumented cranks.

#### Ultrasonography recording equipment

Two B-mode ultrasonography devices (LV7.5/60/96Z, TELEMED, Vilnius, Lithuania) were used to assess VL muscle fascicle length from the right lower limb via two flat transducers mounted in series within a frame. Before the recording, the transducers were covered in conductive gel and in direct contact with the participant's skin. Then, both were placed in series on the mid-thigh, taking as a reference a straight line between the greater trochanter and superior face of the patella (Brennan et al., 2018), and secured using a self-adhesive compression bandage (Fig. 1A). Both ultrasound units were synchronized utilizing analog pulses sent from a separate A/D board that triggered collection of each individual frame (Micro 1401-3; AnySyncro software). The sampling rate was configured and recorded, using Spike 2 software (Cambridge Electronic Design Ltd, Cambridge, UK) through sending a 160 Hz quadratic logic pulse to the ultrasound equipment connected in parallel. This logic pulse also triggered the start of motion-capture collection, allowing an exact synchronization between the probes and ultrasound recordings and the other signals.

#### EMG

Surface EMG data (Myon 320 system; Myon AG, Baar, Switzerland) were collected at 2 kHz using sensors placed on the GM, VL, RF, BFlh and ST of the right leg and were synchronously recorded with motion-capture data via the A/D board controlled by the Qualisys Track Manager Software. The electrodes were adhered to the subjects' skin following SENIAM guidelines with an interelectrode distance of 2 cm (Hermens et al., 2000). Before the placement, the hair over the muscle was shaved with a disposable razor and the skin cleaned with 70% isopropyl alcohol.

### Data analysis

#### Muscle fascicle shortening velocity

Images from the two transducers were concatenated considering a scale of 60 mm and with a space of 22 mm between each field of vision (FOV) of the probes, using a custom MATLAB script (MathWorks Inc.; Fig. 1B). A custom MATLAB script based on the Lucas–Kanade optical flow algorithm was used to track muscle fascicles and measure the changes in VL muscle fascicle length (Gillett et al., 2013; Brennan et al., 2018). The length changes were estimated considering a whole fascicle from the proximal intersection with the superficial aponeurosis and vice versa. The velocity of the VL fascicle with respect to the crank angle was calculated as first derivative of their lengths with respect to time. The fascicle shortening velocity was calculated between the maximum and minimum length within the downstroke phase with respect to crank angle (knee extension period). The peak and average shortening velocities during the downstroke phase were calculated.

#### Joint power analysis

Data obtained from the forces recorded by the pedals of the instrumented crank and by the kinematic variables from 3D motion-capture cameras were exported and analyzed via a custom MATLAB script (R2019a, MathWorks Inc.). All signals were filtered using a zero-lag low-pass second-order Butterworth filter with a 12 Hz cut-off frequency. To represent the task cycle, the crank angle was reconstructed from the right foot kinematic data from a virtual marker representing the pedal axle, considering markers from the lateral and medial ankle and calcaneus. Also, this virtual marker was used to convert the tangential and radial forces into vertical and horizontal components with respect to the global coordinate system. To rotate the force components to the ergometer's reference system, matrix rotation algorithms were applied, giving the resulting pedal forces, which were projected to the pedal axle. Subsequently, an open-source refined model of the lower extremity (Lai et al., 2017) was scaled to each cyclist's anthropometric measurements. Then, the cyclist-specific models, as well as the experimental kinematic and kinetic data were used to compute joint angles and joint moments applying inverse kinematic and inverse dynamics tools in OpenSim (v4.3.0) (Delp et al., 1990). We further calculated joint power as the product of net joint moments and joint angular velocity. Joint work per cycle was calculated as the time integral of joint power per cycle. To calculate joint-specific power, flexion and extension phases were determined from the angular velocity signs. As such, negative specific power values were obtained when the moments and angular velocities were in opposite directions. Joint-specific power values were averaged over each crank cycle (Elmer et al., 2011).

#### Complementary variable analysis

All EMG signals were processed with a 15/500 Hz bandpass filter to remove non-physiological signal noise. To remove the analog channel offset, the signal median activation for each muscle was subtracted. The RMS was calculated with a moving window width of 50 ms as an index of the signal amplitude. Further, the mean EMG signal for each muscle was normalized to the peak EMG RMS value in each cycle during the 90 rpm cadence at 50% *P*_max_. Some VL muscle EMG data were discarded because of sensor displacement elicited by contact with the ultrasound transducers. EMG data collected from GM and RF muscles were averaged across 12 subjects, whereas those for VL, BFlh and ST were averaged across 10 subjects.

To have a better representation of the changes in joint moments, the averaged value of the knee joint moment in the positive extension phase was considered. The positive extension phase was calculated by determining the period of positive knee angular velocity for each cadence condition. The lengths and velocities of the VL MTU were calculated using the inverse kinematics results and the muscle analysis tool in OpenSim.

### Statistical analysis

All values were taken as the average of the first five cycles of the right crank per each cadence condition and crank power requirement (note, the crank angle at top-dead-center was defined as 0 deg). A two-way repeated measures ANOVA was used to test for main effects of cadence and crank power requirements and interaction effects on muscle shortening velocity, joint power, positive knee extensor moments and EMG signal. A repeated measures one-way ANOVA was used to test the main effect of the crank power requirement at SSC on muscle shortening velocity. Tukey multiple comparisons tests were made across cadence conditions within each level of analysis. To quantify how much more likely the muscle shortening velocities at SSC across crank power requirements are under the null hypothesis, we performed Bayesian ANOVA analyses, considering a credible interval of 95% and *post hoc* tests. The Bayes factor towards the null hypothesis was denoted as BF_01_. For its interpretation, for BF_01_>1 and <3 there is anecdotal evidence, for BF_01_>3 and <10 there is moderate evidence, for BF_01_>10 and <30 there is strong evidence, for BF_01_>30 and <100 there is very strong evidence and for BF_01_>100 there is extreme evidence towards the null hypothesis. The EMG data were also fitted through a second-order polynomial non-linear regression to examine the relationship between the mean EMG RMS activation and cadence at each pedal power requirement. The coefficient of determination (*R*^2^) was used to compare the experimental and predicted SSC in which the minimum EMG RMS was elicited across muscles and pedal power requirements. An α level of 0.05 was set for all tests. All data were processed using Prism 7 (GraphPad Software Inc., La Jolla, CA, USA) and JASP (https://jasp-stats.org/).

## RESULTS

During the force–velocity test, participants performed a total averaged peak power of 898±37 W. This yielded power targets at 10% (89.8±3 W), 30% (270±10 W) and 50% (449±18 W) *P*_max_. The self-selected cadence protocol showed that cyclists increased their cadences as crank power increased (e.g. 30% versus 50% *P*_max_ *P=*0.001), reaching cadences of 94±11, 103±11 and 117±12 rpm at 10%, 30% and 50% *P*_max_, respectively. Table 1 presents comprehensive statistical information (*F*, *P*, η^2^) regarding the effects of crank power requirement and cadence and their interactions on muscle shortening velocity, joint power, positive knee extensor moments and EMG signal.

**Table 1.**
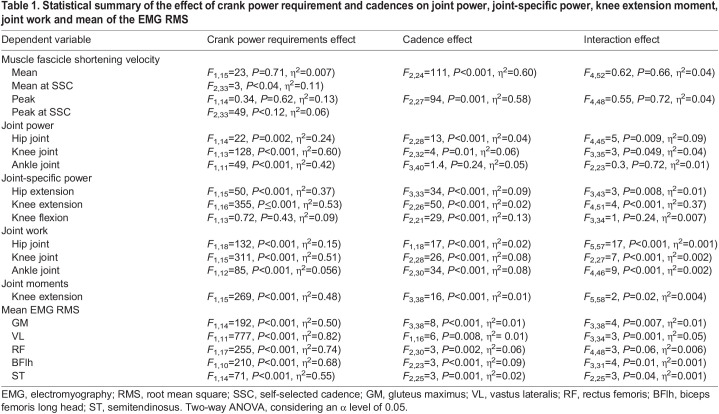
Statistical summary of the effect of crank power requirement and cadences on joint power, joint-specific power, knee extension moment, joint work and mean of the EMG RMS

### Muscle fascicle shortening velocity

**Fig. 2.**
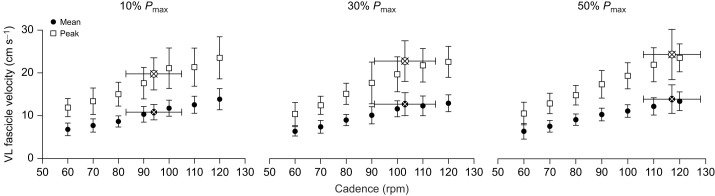
**VL fascicle shortening velocity at different cadences and external power requirements.** Mean and peak absolute values (±s.d.) at 60, 70, 80, 90, 100, 110 and 120 rpm and the self-selected cadence (SSC) for 10%, 30% and 50% *P*_max_ are shown (where SSC was 94 rpm at 10% *P*_max_, 103 rpm at 30% *P*_max_ and 117 rpm at 50% *P*_max_, indicated by a cross for both means and peaks).

There was no main effect of pedal power requirements on mean fascicle shortening velocity (Table 1). There was a significant effect of cadence on mean fascicle shortening velocity, with mean fascicle shortening velocity increasing with cadence in a similar manner across all crank power requirements (Table 1; Fig. 2). The increase in mean fascicle shortening velocity with cadence was approximately 1.01, 0.92 and 1.16 cm s^−1^ per 10 rpm at 10%, 30% and 50% *P*_max_, respectively. There was no interaction effect between pedal power requirements and cadence on mean fascicle shortening velocity (Table 1).

There was a significant main effect of pedal power requirement on mean fascicle shortening velocity at SSC (Table 1; 10% *P*_max_: 10.8±1.8 cm s^−1^; 30% *P*_max_: 12.5±2.5 cm s^−1^; 50% *P*_max_: 13.5±3.1 cm s^−1^). *Post hoc* comparison showed a significant increase in fascicle velocity between 10% and 50% *P*_max_ (*P*=0.04), but not between 10% and 30% *P*_max_ (*P*=0.26) and 30% and 50% *P*_max_ (*P*=0.61) (Fig. 2). The Bayesian ANOVA *post hoc* analysis showed anecdotal evidence inclined to *H*_0_ when evaluating mean shortening velocity at SSC (e.g. 10% versus 30% *P*_max_: BF_01_=0.8; 30% versus 50% *P*_max_: BF_01_=2).

There was also no significant effect of pedal power requirement on peak fascicle shortening velocity (Table 1; Fig. 2). There was a significant effect of cadence on peak fascicle shortening velocity, with shortening velocity increasing with increasing cadence (Fig. 2). The was no significant interaction effect between crank power requirement and cadence on peak fascicle shortening velocity (Table 1).

There was no significant main effect of pedal power requirement on peak fascicle shortening velocity at SSC (Table 1). Modest evidence in favor of *H*_0_ was found on peak shortening velocities at SSC across pedal power requirements (e.g. 10% versus 30% *P*_max_: BF_01_=1.2; 10% versus 50% *P*_max_: BF_01_=0.5; and 30% versus 50% *P*_max_: BF_01_=2).

### Joint power and mechanical work

**Fig. 3.**
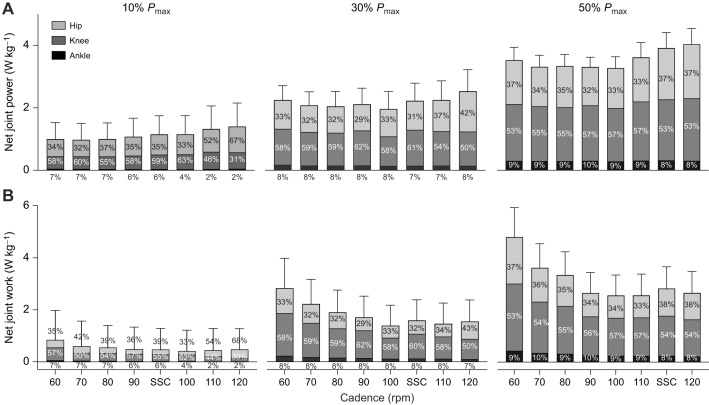
**Joint power and joint work distribution at different cadences and external power requirements.** Mean (±s.d.) joint power (A) and work (B) at 60, 70, 80, 90, 100, 110 and 120 rpm and SSC for 10%, 30% and 50% *P*_max_ are shown (where SSC was 95 rpm at 10% *P*_max_, 103 rpm at 30% *P*_max_ and 113 rpm at 50% *P*_max_).

**Fig. 4.**
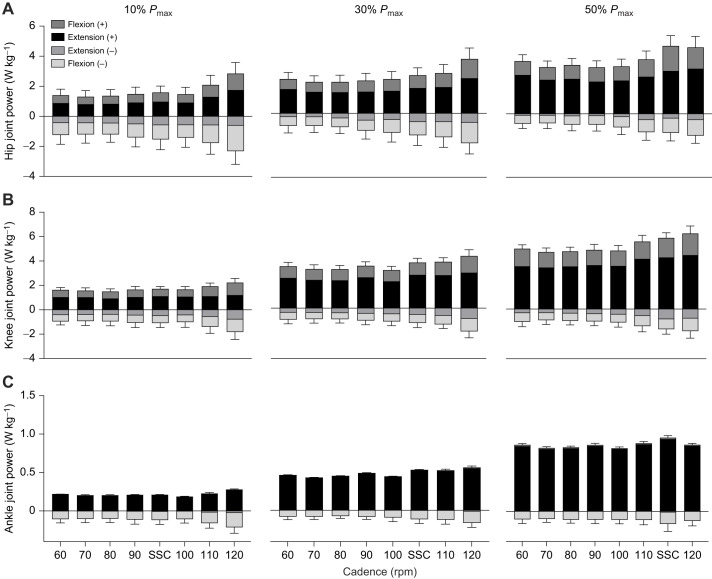
**Decomposition of joint power into positive and negative contributions during net flexor and extensor muscle power at different cadences and external power requirements.** Mean (±s.d.) hip (A), knee (B) and ankle (C) joint power at 60, 70, 80, 90, 100, 110 and 120 rpm and SSC for 10%, 30% and 50% *P*_max_ are shown (where SSC was 95 rpm at 10% *P*_max_, 103 rpm at 30% *P*_max_ and 113 rpm at 50% *P*_max_).

The power output for each joint relative to the total summed power across joints (for all crank power requirements and cadence conditions) is shown in Fig. 3A, whilst the individual contributions of positive and negative power for flexion and extension phases are shown for the hip, knee and ankle in Fig. 4A–C. Fig. 3B illustrates the total summed mechanical work of each joint across all cadences and crank power requirement conditions.

A main effect of crank power requirement, cadence and an interaction effect (crank power requirements×cadence) were found for the relative contribution of hip and knee joint power to total power. Ankle joint power was affected by crank power requirement, but there was no cadence or interaction effect (Table 1). All joints increased their power contribution with increased crank power requirement (Fig. 3A). A main effect of cadence shows that there was a greater summed power output across hip and knee joint power at higher cadences. However, this was mainly constrained to cadences above 110 rpm (e.g. hip joint power at 90 rpm: 0.37, 0.70, 1.17 W kg^−1^; versus 110 rpm: 0.53, 0.85, 1.30 W kg^−1^: *P=*0.01, *P=*0.02 and *P=*0.01 at 10%, 30% and 50% *P*_max_, respectively; Fig. 3A). Breaking down power into flexion and extension components revealed similar trends: hip extension, knee extension and knee flexion power (Table 1; Fig. 4) increased in unison as power increased.

A significant main effect of crank power requirement, cadence and their interaction was found for hip, knee and ankle joint work (Table 1). A significantly larger work per cycle was found at low cadences across hip and knee joints (e.g. knee joint work at 60 rpm: 0.48, 1.64, 2.50 J kg^−1^; versus 80 rpm: 0.29, 1.13, 1.84 J kg^−1^; *P=*0.004, *P=*0.001 and *P=*0.001 at 10%, 30% and 50% *P*_max_, respectively; Fig. 3B). Nonetheless, the distribution of work across the three joints remained similar across cadences for each of the power conditions tested.

### Complementary variables

**Fig. 5.**
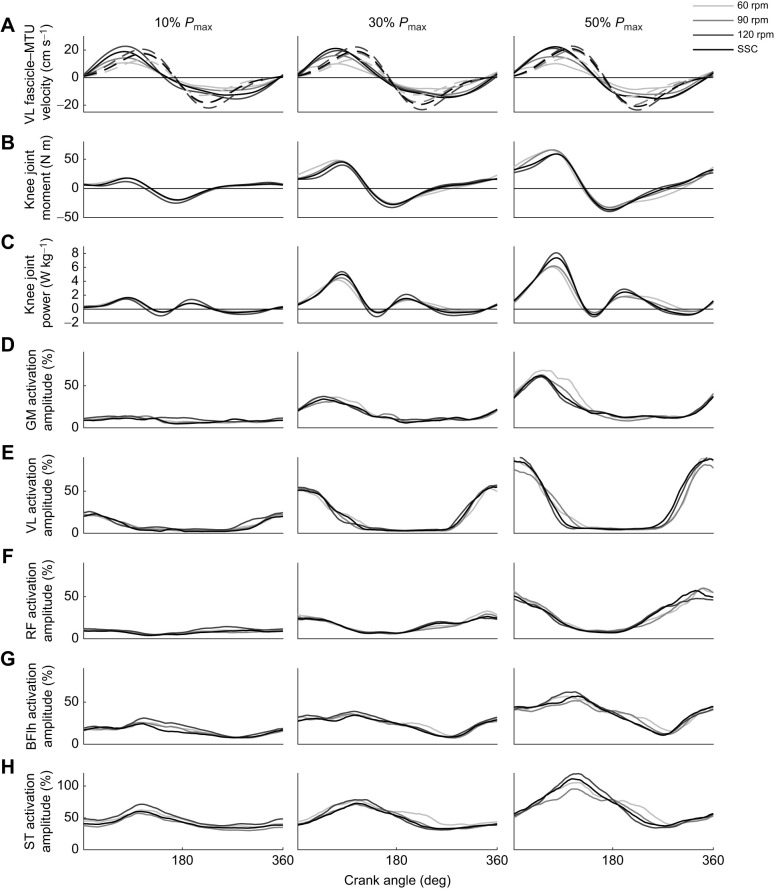
**Mean waveforms of knee muscle mechanics and activation and gluteus maximus activation.** (A) Mean VL fascicle (solid lines) and muscle–tendon unit (MTU; dashed lines) shortening velocity. (B,C) Knee joint moments (B) and power (C). (D–H) Muscle EMG activation from gluteus maximus (GM; D), VL (E), rectus femoris (RF; F), biceps femoris long head (BFlh; G) and semitendinosus (ST; H). All waveforms were plotted across 60, 90 and 120 rpm and SSC, at 10%, 30% and 50% *P*_max_ against the crank angle cycle. The crank angle is 0 at top-dead-center. The s.d. and remaining cadences (70, 80, 100, 110 rpm) were omitted for clarity.

There was a main effect of crank power requirement, cadence and their interaction on positive knee extensor moments (Table 1), showing a systematic decrease as the cadence increased across all crank power output conditions (e.g. at 30% *P*_max_, 25.5±10, 23.9±10 and 23.2±10 N m for 60, 70 and 80 rpm versus 18.4±10 N m at 120 rpm; *P=*0.001, *P=*0.004 and *P=*0.001, respectively; Fig. 5B).

**Fig. 6.**
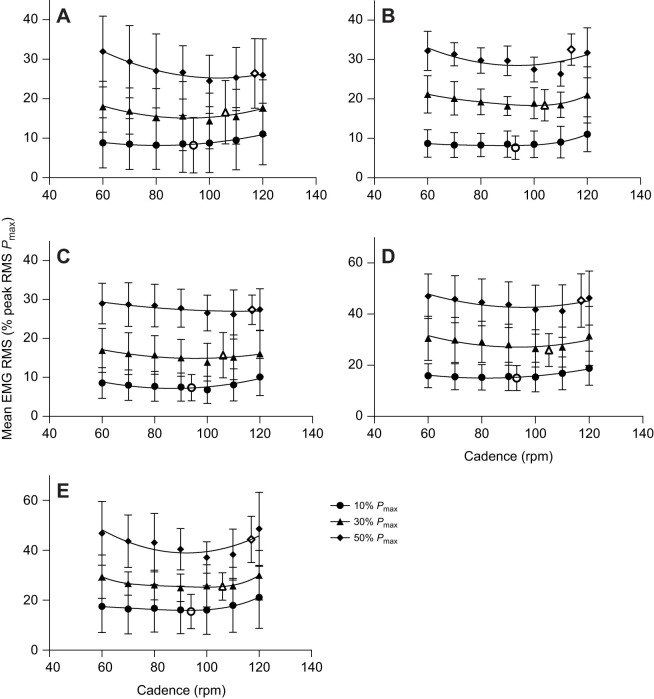
**Mean EMG root mean square (RMS) of GM and knee muscles at different cadences and external power requirements.** Mean (±s.d.) GM (A), VL (B), RF (C), BFlh (D) and ST (E) EMG RMS at 60, 70, 80, 90, 100, 110, 120 rpm and SSC for 10%, 30% and 50% *P*_max_ are shown (where SSC was 94 rpm at 10% *P*_max_, 106 rpm at 30% *P*_max_ and 117 rpm at 50% *P*_max_, represented by open symbols). Lines represent a second-order polynomial non-linear regression.

**Fig. 7.**
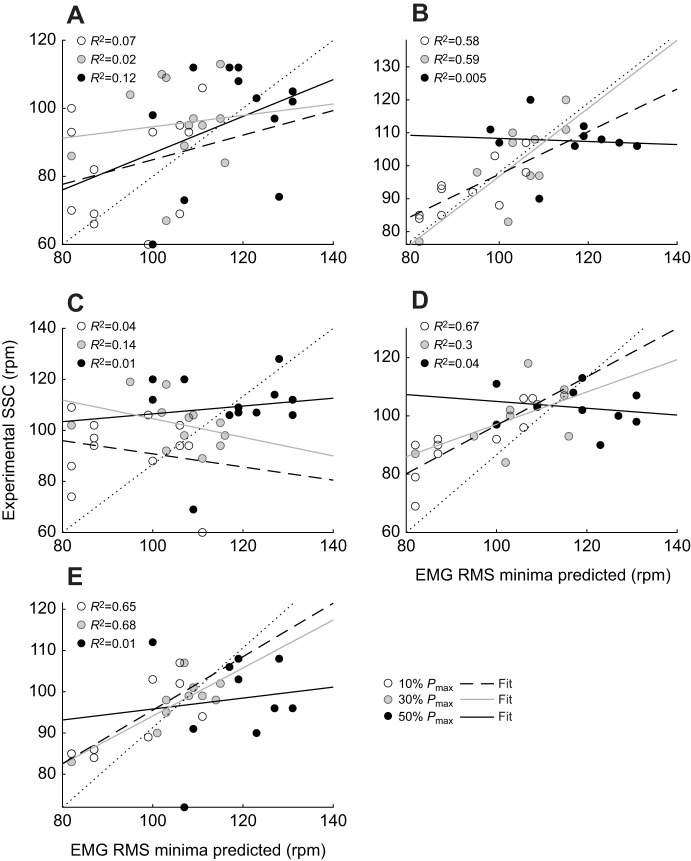
**Linear regression and *R*^2^ coefficients between experimental SSC and predicted values.** EMG RMS minima were predicted from the non-linear regression per subject across GM (A), VL (B), RF (C), BFlh (D) and ST (E) at 10%, 30% and 50% *P*_max_. Lines indicate the fit per pedal power demands (see key).

A significant main effect of crank power requirement, cadence and their interaction was found for the mean EMG RMS for the GM, VL, BFlh and ST muscles (Table 1). RF was only affected by crank power requirements and cadence. The mean EMG RMS signal of all muscles increased as power pedal requirements increased (*P=*0.001). Changes in muscle activation with cadence for different crank power requirements were non-linear for all muscles analyzed (Fig. 6). The non-linear regression revealed a ‘U’-shaped relationship between the mean EMG RMS and cadence for all muscles, showing a minimum at higher cadences as the power demands increased (Fig. 6). Furthermore, the mean EMG RMS activation at the SSC showed closeness to those where the local minima were predicted from the non-linear regression for VL (*R*^2^=0.58 at 10% *P*_max_; *R*^2^=0.59 at 30% *P*_max_), BFlh (*R*^2^=0.67 at 10% *P*_max_ only) and ST (*R*^2^=0.65 at 10% *P*_max_; *R*^2^=0.68 at 30% *P*_max_) muscles at 10% and 30% *P*_max_ (Fig. 7).

## DISCUSSION

This study examined changes in VL fascicle shortening velocity and joint-specific power contributions during cycling at a range of power outputs and cadences. We found that the net fascicle shortening velocity at a fixed range of cadences was not affected by changing the crank power requirement. Hence, fascicle velocity requirements largely mirror those of the MTU, which are constrained by the prescribed cadence. Our results also showed an increase in mean muscle shortening velocity at the SSC as the power requirement increased. This suggests that cadence selection is not driven by the desire to maintain a single, ‘unique’ shortening velocity that maximizes power capacity regardless of the intensity exerted as previously conceptualized by Brennan et al. (2019). However, the observations may still be consistent with a desire to recruit muscles close to their optimal shortening velocity for power production, assuming greater recruitment of fast-twitch motor units as power demands increase. Unfortunately, because of complex lateral interactions between muscle fibers within individual and groups of muscles (Bottinelli, 2001; Talmadge et al., 1993; Holt et al., 2014) and individual differences in fiber-type proportions (Coyle et al., 1992; Simoneau and Bouchard, 1989), it is difficult to predict the magnitude of expected changes in optimal shortening velocity with cycling power demands. Thus, the role of fascicle shortening velocity in the selection of cycling cadences remains an open question. Our data also demonstrated that the power and work output of each joint increased as required, but showed little effect on their redistribution from one joint to another as pedal power requirements increased. This indicates that there was little change in motor coordination strategy across the range of power requirements studied here. Finally, we also found that the mean EMG RMS activation of knee muscle at the SSC was close to a local minimum at low pedal power demands, providing further evidence that the drive required to the muscle may be a key factor in SSC selection. Therefore, our findings support previous conclusions (MacIntosh et al., 2000; Takaishi et al., 1996) that minimization of muscle activation may be an important factor that drives the SSC.

While fascicle shortening velocity in pre-set cadence conditions was not affected by changes in pedal power requirements, it increased progressively as cadence increased regardless of the power demand exerted. Although this is contrary to Brennan et al.’s (2019) observation of a plateau behavior in shortening velocity at cadences beyond 80 rpm, our results confirm previous observations of a decoupling between MTU and fascicle velocity during the early phase of the crank cycle, specifically when the force is rising (Fig. 5A) (Muraoka et al., 2001; Brennan et al., 2018). These responses might be due to the influence of the elastic component of the tendinous tissue for force development (Muraoka et al., 2001). Furthermore, during the cadence selected by riders (SSC), we found that VL fascicle shortening velocity increased as crank power requirements increased. Because SSC increased to higher cadences as power demands increased, this required an increase in VL fascicle shortening velocity, as reflected in our fixed cadence data. For example, SSC increased from 94±11 to 117±12 rpm, in unison with the increase in mean fascicle shortening velocity from 10.85 to 13.5 cm s^−1^, between 10% and 50% *P*_max_, respectively (Fig. 2). Thus, it appears that there is no unique muscle shortening velocity that underpins a desire to maintain optimal levels for maximal power generation. By contrast, previous findings suggested selecting high cadences at high power outputs may reduce the required force, generating a negligible impact on the net fascicle shortening velocity because of the series elastic structure involvement. The proposal was that this might allow the maintenance of stable fascicle shortening velocities (Brennan et al., 2019). Our results do not support this idea, although an expected increase in twitch fiber recruitment at higher power outputs leaves open the possibility that fascicle shortening velocity at the self-selected cadence was approximately matched to the optimal for power production capacity.

The increased VL fascicle shortening velocity with cadence reduces the force-generating potential of the muscle; however, increasing cadence also reduced the knee extensor force requirements in all power conditions, and these two factors likely trade-off to influence the required VL muscle activation. Our VL EMG results show different interactions between cadence and activation under each power condition. As such, the U-shaped relationship between cadence and activation across power output conditions (Fig. 6) had a minimum that occurred at progressively increasing cadences, in line with shifts in the SSC and the mean VL fascicle shortening velocity. This may relate to the hypotheses proposed by Sargeant (1994) and MacIntosh et al. (2000) based on indirect measurement of the relationship between force and velocity of muscle shortening using a cycle ergometer (i.e. the resistance–velocity relationship) and EMG. They calculated that because of the additional recruitment of fast-twitch fibers, the optimal muscle shortening velocity, and hence the minimum level of muscle activation, should occur at a higher cadence with increases in cycling power demands. Whilst this might help explain how both activation and power-generating potential can be optimized with increased cadence at progressively increasing power output requirements, further work is required to confirm that the fascicle velocity at which power is optimized increases with increased force (resistance) and power output. We found only one study demonstrating such a phenomenon in a large, mixed fiber type muscle: the human wrist flexors (Chow and Darling, 1999). Thus, given that our current analysis of the mean fascicle shortening velocity for a cadence at different power outputs is restricted to the VL muscle, further simulation is required to understand the relationship between fascicle velocity and muscle activation on a larger scale.

Contrary to our expectation, we found that there was little change in power distribution across joints with changes in pedal power demands across the cadence range. This result contrasts with that of Elmer et al. (2011), who found a redistribution at higher power outputs; specifically, a drop in knee extensor power (4%) and increase in hip power (4%). We cannot pinpoint the reasons for discrepancies; however, the study by Elmer et al. (2011) did test higher power outputs (up to 550 W), which might explain the differences. Further, with this negligible distribution, it appears that knee extensor power contribution is not affected by pedal power demands in the range of power outputs studied here, which has implications for interpretations of other variables. Although the knee extension force (as knee extension moment) decreased as the cadence increased (Fig. 5B), it increased from 23 to 34 N m between 30% and 50% of the *P*_max_ at 90 rpm. Thus, this suggests that it is unlikely that knee extensor power may be limited by force generation capacity.

Our results suggest that there may also be some further negative mechanical effects in selecting higher cadences as pedal power requirements increase. We found that at high cadences (>100 rpm), there was an increase in the net joint power production which was mainly attributed to the power from hip and knee joints (Figs 3A and 4A,B). This is likely due to the fact that faster limb movements require the generation of additional segmental kinetic energy, hence increasing the internal work generated to control higher segmental accelerations at faster cadences (Kautz and Neptune, 2002). However, high cadences also affect the cyclist's ability to effectively accelerate the crank because of the reduction in time available for activation and deactivation of muscles (Fig. 5D–H), which can lead to an increase of negative muscular work (Neptune and Herzog, 1999a). From our results, this can be extrapolated through the analysis of knee joint power across the cycle (Fig. 5C), specifically at the end of the upstroke phase in the crank cycle, where there is an increase in the knee negative power. In order to generate required knee extensor forces at the top of the crank cycle, our VL EMG data show it is necessary to activate the VL muscle earlier relative to the crank position (Fig. 5E). As such, the knee extensor moments increased earlier in the upstroke as cadence increased (Fig. 5B), contributing to greater absorption of energy at the knee.

In line with previous research (MacIntosh et al., 2000), our study showed that muscles other than the VL (GM, BFlh, ST and RF EMG RMS) also exhibited a U-shaped relationship with cadence, with the minimum shifting to higher cadences as the crank power requirement increased (Fig. 6). These results suggest that it might be convenient to select higher cadences as the power demands on the pedal increase in order to maintain minimum muscle activation. VL, BFlh and ST muscles were shown to be close to minimum EMG RMS at the SSC at 10% and 30% *P*_max_ (Figs 6 and 7). By contrast, although the SSC was larger at 50% *P*_max_ than at 10% and 30% *P*_max_, it exceeded the cadence with the minimum EMG RMS activation in all the muscles assessed (Figs 6 and 7). This finding might suggest that the cadence selected by riders at submaximal power output (10% and 30% *P*_max_) may be a strategy to minimize fatigue, as previous studies suggest in simulated (Neptune and Hull, 1999b) and endurance cycling (Takaishi et al., 1996). These responses may be seen as the nervous system prioritizing the minimization of activation of overburdened muscles to prolong the movement duration (McDonald et al., 2022 preprint). Besides, there may also be a reduction in peak pedal forces (Hagberg et al., 1981), which would physiologically improve blood flow as a result of the reduction in intramuscular pressure and thus mitigate fatigue (Hagberg et al., 1981; Takaishi et al., 1998). In contrast, at very high power outputs, other factors such as minimizing force requirements may be more preferential.

### Limitations

Certain limitations should be acknowledged in our study. Only men were recruited to reduce the outcome variability. It has been shown that male muscles have higher power output and faster muscle groups (Glenmark et al., 2004), and hence are likely to have different muscle fascicle shortening velocities. The SSC used as a preset value on the second day of assessment was based on the results obtained during the first day. Although SSC might be affected on the second day, the available evidence indicates that SSC is minimally affected by internal and external conditions during cycling (Hansen and Ohnstad, 2008). Further, conditions such as ergometer configuration and power requirements were maintained on both days.

Our measurements of VL fascicle length and velocity based on ultrasound images have some potential limitations. For example, muscle deformation and horizontal shifting of connective tissues can cause an underestimation error of the fascicle length given by the muscle deformation (Brennan et al., 2017) (e.g. the fascicle origin and insertion); a greater horizontal shifting was observed for connective tissues in parallel (e.g. superficial, and deep aponeurosis) at lower crank power requirements (10% *P*_max_), which may affect fascicle endpoint estimation. To counter this, aside from including ultrasound dual probes, the VL fascicle tracking was optimized by increasing the sampling rate to 160 Hz. This is because higher sample rates perform better for faster movements such as cycling as a result of the maximization in time resolution, enhancing fascicle tracking (Cronin and Lichtwark, 2013). Thus, the sampling rate selected likely improved the measurement of data extracted from the muscle fascicle.

### Conclusion

We found that while net fascicle shortening velocity in a preset range of cadences was not affected by changing the crank power requirement, there was an increase in shortening velocity at the SSC as power output increased. This is in contrast with a previous report that shortening velocities were consistent with maximal power production at the SSC during low power output. Further, changes in joint power distribution were not observed in submaximal to maximal cycling transitions. Instead, cyclists selected higher cadences, which may reduce force requirements and possibly maintain fascicle velocities close to the optimal for power generation as a result of increased recruitment of fast-twitch fibers. At low power demands, our results suggest that minimization of muscle activation (EMG RMS) in the knee may be a component to consider when selecting a cadence. Thus, these findings highlight the important differences in mechanical and muscular activation responses to changes in power pedal demands during cycling, suggesting that muscle activation may be a critical aspect for riders in selecting their cadences, but the performance criterion that governs bicycling tasks remains unclear.
